# Special Issue “Nanoplatforms Based Cancers Therapy”

**DOI:** 10.3390/cancers15061698

**Published:** 2023-03-10

**Authors:** Magali Gary-Bobo

**Affiliations:** IBMM, University of Montpellier, CNRS, ENSCM, 34093 Montpellier, France; magali.gary-bobo@umontpellier.fr

Nanomedicine is now considered a hopeful strategy to efficiently target cancer cells and deliver, more specifically, the molecule of interest to the area to image and treat cells. A large variety of biocompatible nanoparticles has been produced in the last few years to carry and protect from biological barriers the molecules designed for imaging or therapy. In addition, a stimuli response system such as laser, temperature, ultrasounds, etc., to deliver the cargo at the site selected, has shown to bring a supplementary dimension for the conception of high-potency controlled nanodevices. Another crucial interest of the nanodevice is the high loading capacity to detect molecules of interest and the ability to be anchored with targeting molecules to efficiently address the nanoplatforms to the tumor area and, more particularly, to cancer cells. This Special Issue, constituted by four original articles and three reviews, focuses on innovative nanoplatforms for the therapy and imaging of cancers.

The first original contribution concerns the elaboration of magnetic cobalt ferrite spinel (MCFS) nanoparticles with unique double-contrast properties enabling effective T1-weighted and T2-weighted magnetic resonance imaging (MRI) [1]. Importantly, these MCFS nanoparticles are efficient for in vivo MRI applications, which are more than most common contrast agents. MCFS nanoparticles were injected in mice and a toxicity study was performed. The structural and functional integrity of kidney, liver, pancreas, and heart were verified, and the safety of these nanoparticles was established. These MCFS nanoparticles can also become a targeted drug delivery system; in addition, they exhibit simultaneous T1- and T2-weighted MRI properties. For this, liposomes encapsulating MCFS nanoparticles and doxorubicin hydrochloride were produced. When encapsulated in PEGylated liposomes loaded with doxorubicin, the efficiency in mice was higher than doxorubicin itself to decrease tumor size. This study demonstrates the potential of the MCFS nanoparticles for a variety of therapeutic and imaging applications.

Several articles have demonstrated the advantage of liposomes for drugs addressing the area of interest, and in this second article [2], lipidic nanoparticles were loaded with a prodrug (docetaxel) and with gold nanoparticles. The results were very promising as these nanoparticles loaded with docetaxel have a superior targeting of tumor tissues compared to free drug. Importantly, these nanoparticles containing gold nanoparticles exhibited minimal toxicity to normal tissues and an ideal radiosensitization property useful for radiotherapy. Altogether, the data demonstrated synergistic therapeutic effects.

In the same way, the advantages of nanomedicine for cancer targeting were well described in a first review published by Foglizzo and Marchiò [3] who explained why nanoparticles optimize drug delivery to the site of interest by reducing the accumulation in healthy tissues. The use of nanoparticle formulations allows for a selective cancer cells death while avoiding affecting all the cellular network in the body and deleterious side effects. This review focused on their physicochemical properties and targeting functionalization, leading to a high efficiency for cancer therapy. Importantly, this review explains how this strategy of nanoplatform, combining different therapies and targeting moieties in a single formulation, can target cancer, overcome multidrug resistance, and also be a breakthrough tool for cancer vaccines and immunotherapy.

In addition, another way of thinking about the improvement of tumor targeting for nanoparticles consists of modifying the tumor site and its vascularization. In a second review [4], different strategies applied by researchers to improve the nanomedicine penetration to tumors, taking into account the complexity of cancer micro-environments responsible for delivery problems, were explored. Several solutions have been proposed to increase nanomedicine access to tumors, such as the manipulation of the tumor micro-environment. This is an original opinion in which the modulation of the tumor extracellular matrix and blood vessels that change the tumor microenvironment may be as important as nanoparticles’ characteristics (size, potential zeta, shape, etc.) to improve the penetration and accumulation of nanoparticles inside tumor site. For example, therapies such as radiations, or hyperthermia, could modify the blood vessels of neoangiogenesis in a part, and blood flow in another part, leading to a significant accumulation of nanoparticles in general and liposomes containing drugs in particular. This review helps increase the awareness of the importance of cancer pathophysiology and tumor micro-environment knowledge.

This is also the case in the following article, that concerns the description of the anti-cancer mechanism of a synthetic inhibitor of KRAS gene (KR12), containing pyrrole-imidazole polyamide (PIP), which is well known to induce tumor accumulation. In this study performed by Higashi et al. [5], the complex contains a PIP part that addresses KR12 to the KRAS gene for an efficient blocking. This induces a remarkable reduction in tumor size with a well-described mechanism of action. Indeed, the moieties constituting this nanocomplex induce a preferentially tumor accumulation, while no nanocomplex was detectable in brain, lung, stomach, kidney, spleen, liver, intestine, and heart. In vivo experiments were performed on the CAM model xenografted with human cancer cells, in which a strong reduction in tumor size was demonstrated when nanocomplexes were injected. This anti-cancerous effect was also correlated with a time-dependent and dose-dependent accumulation of nanocomplexes in the nucleus of cancer cells thanks to the PIP part. In this article, we can see the interest of nanomedicine when genetic and physiology around the cancer to treat is well mastered.

Another concept for nanoparticles, apart from drug delivery, is the encapsulation of photoactivable molecules into biocompatible nanovectors. This is particularly interesting for hydrophobic porphyrins with highly luminescent and photoactive properties to generate singlet oxygen in organic solvents, but which lost all their advantages in biological and hydrophilic medium. This is the goal of the last original article of this Special Issue [6] in which biocompatible and polymeric nanoparticles have been tested as nanocarriers to water-solubilize star-shaped hydrophobic porphyrins which have been shown to be efficient for theranostic approaches under a two-photon excitation laser in the near infrared area. Indeed, this work describes the photodynamic therapy (PDT) efficiency to kill cancer cells and the luminescence emission for bioimaging purposes, in spite of their confinement in the nanoparticles, validating definitely the use of these nanovectors for theranostic purposes.

The use of a laser or lamp is becoming increasingly popular to stimulate active principles encapsulated in nanoparticles in order to generate singlet oxygen production and luminescence emission for PDT and imaging, respectively, but also for another mechanism, which is called photochemical internalization (PCI). The PCI mechanism consists of favoring the transfer of material from lysosomes to cytoplasm via lysosomal escape induced by lysosomal membrane destabilization under laser excitation. The last review, [7], focused on the different nanovectors capable of transporting genetic material such as small-interfering RNA (siRNA) in order to block the expression of genes responsible for the development of cancer. Because the nanoparticles are mainly internalized via the endolysosomal pathway, and the RNA is found in the cytoplasm, the PCI mechanism is fully suitable for siRNA action, of which lysosomal escape is laser induced. 

Finally, we hope this Special Issue may have drawn attention to the large panel of nanovectors and the tremendous field for various biomedical applications. Indeed, nanoparticles can be composed by various components, materials, and loading several active principals (drugs, nucleic acids, photosensitizers, dye, metals, etc.). These are hybrid and complex nanomaterials which can also be anchored with targeting molecules for a custom design and a total control in the synthesis, which is elaborated for best tumor accumulation.

## References

[1] Mikhaylov G., Mikac U., Butinar M., Turk V., Turk B., Psakhie S., Vasiljeva O. (2022). Theranostic Applications of an Ultra-Sensitive T1 and T2 Magnetic Resonance Contrast Agent Based on Cobalt Ferrite Spinel Nanoparticles. Cancers.

[2] Alhussan A., Jackson N., Eaton S., Santos N.D., Barta I., Zaifman J., Chen S., Tam Y.Y.C., Krishnan S., Chithrani D.B. (2022). Lipid-Nanoparticle-Mediated Delivery of Docetaxel Prodrug for Exploiting Full Potential of Gold Nanoparticles in the Treatment of Pancreatic Cancer. Cancers.

[3] Foglizzo V., Marchiò S. (2022). Nanoparticles as Physically- and Biochemically-Tuned Drug Formulations for Cancers Therapy. Cancers.

[4] Munir M.U. (2022). Nanomedicine Penetration to Tumor: Challenges, and Advanced Strategies to Tackle This Issue. Cancers.

[5] Higashi Y., Ikeda S., Matsumoto K., Satoh S., Komatsu A., Sugiyama H., Tamanoi F. (2022). Tumor Accumulation of PIP-Based KRAS Inhibitor KR12 Evaluated by the Use of a Simple, Versatile Chicken Egg Tumor Model. Cancers.

[6] Shi L., Nguyen C., Daurat M., Richy N., Gauthier C., Rebecq E., Gary-Bobo M., Cammas-Marion S., Mongin O., Paul-Roth C.O. (2022). Encapsulation of Hydrophobic Porphyrins into Biocompatible Nanoparticles: An Easy Way to Benefit of Their Two-Photon Phototherapeutic Effect without Hydrophilic Functionalization. Cancers.

[7] Ali L.M.A., Gary-Bobo M. (2022). Photochemical Internalization of siRNA for Cancer Therapy. Cancers.

